# Primary Septic Arthritis of the Hip Caused by Neisseria meningitidis Serogroup W135 in an Infant

**DOI:** 10.7759/cureus.107182

**Published:** 2026-04-16

**Authors:** Hatim Jabri, Meryem Fettah, Mohammed Tazi Charki, Hicham Abdellaoui, Karima Atarraf, Moulay Abderrahmane Afifi

**Affiliations:** 1 Department of Pediatric Orthopedics and Traumatology, Hassan II University Hospital, Fez, MAR; 2 Laboratory of Human Pathology, Biomedicine and Environment, Faculty of Medicine, Pharmacy, and Dental Medicine. Sidi Mohamed Ben Abdellah University, Fez, MAR

**Keywords:** hip, infant, neisseria meningitidis, septic arthritis, serogroup w135

## Abstract

Septic arthritis in children is a medical and surgical emergency, most commonly caused by Staphylococcus aureus. Primary septic arthritis due to Neisseria meningitidis is extremely rare; the absence of meningeal signs often leads to significant diagnostic and therapeutic delay. We report a rare case of primary hip arthritis caused by Neisseria meningitidis serogroup W135 in an infant.

A six-month-old infant with no significant medical history presented with fever and right hip pain following a flu-like syndrome. Clinical examination revealed a pseudoparalytic right limb and a fever of 39°C. Laboratory tests showed elevated inflammatory markers. While pelvic X-rays were normal, ultrasound confirmed a right hip joint effusion. Surgical arthrotomy was performed, yielding purulent fluid that identified Neisseria meningitidis serogroup W135. The patient received dual intravenous antibiotic therapy based on ceftriaxone and gentamicin, followed by oral cefixime. An immunodeficiency workup (HIV, immunoglobulins, and complement levels) was negative. After seven days, the patient showed significant clinical improvement and was discharged from the hospital. Monthly follow-up confirmed a full recovery.

Although rare, Neisseria meningitidis should be considered a potential pathogen in pediatric septic arthritis. Early surgical drainage combined with appropriate antibiotic therapy ensures a favorable prognosis and prevents long-term joint sequelae.

## Introduction

Septic arthritis in children is a medico-surgical emergency, with an estimated incidence between four and 37 cases per 100,000 children [1]. While Staphylococcus aureus is the most frequently implicated pathogen [2,3], arthritis caused by Neisseria meningitidis remains rare, accounting for approximately 1.5% of pediatric cases [2]. Primary meningococcal arthritis is defined as an acute joint infection occurring without meningitis or meningococcal sepsis-a presentation that often poses significant diagnostic and therapeutic challenges [4]. Meningococcal disease may be the underlying cause of what initially appears to be ordinary septic arthritis. Among the 12 identified capsular serogroups of N. meningitidis, serogroup W historically accounted for only a minority of cases. However, after the 2000 outbreak in the Arabian Peninsula, researchers observed a global surge in infections associated with this serogroup [2]. Here, we present the third reported case worldwide of primary hip arthritis due to N. meningitidis serogroup W135 in an infant.

## Case presentation

The patient was a six-month-old infant with no significant medical history and no reported contact with individuals suffering from meningococcal disease. Two days prior to admission, he presented with a flu-like syndrome, characterized by fever and nasal congestion. He was ultimately admitted for persistent fever and pain during mobilization of the right hip. Clinical examination upon admission revealed a conscious infant, hemodynamically and respiratory stable, febrile to 39°C, without signs of meningeal syndrome. Examination of the musculoskeletal system revealed a pseudoparalytic appearance of the right lower limb, with pain upon hip mobilization.

**Figure 1:**
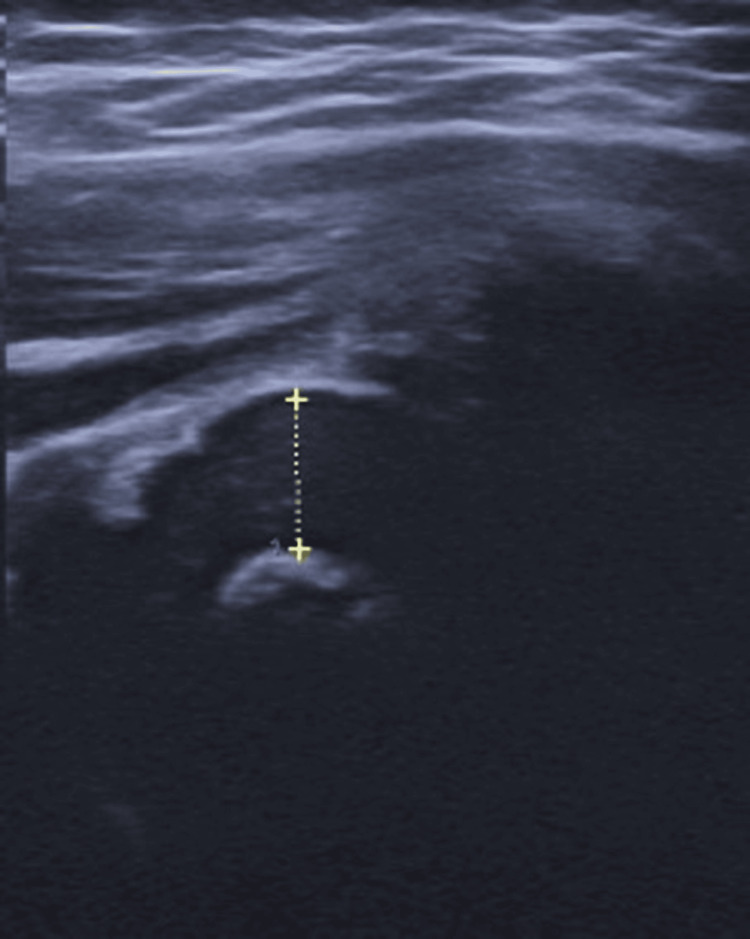
Ultrasound scan of the right hip showing fluid (yellow dots) in the hip joint

Laboratory tests showed a hemoglobin level of 11.6 g/dL, an elevated white blood cell count characterized by a predominance of neutrophils. Additionally, both the C-reactive protein (CRP) and the erythrocyte sedimentation rate were significantly increased, suggesting a systemic inflammatory response (Table 1), while blood cultures were negative. Chest and pelvic X-rays were unremarkable, while ultrasound revealed an echogenic joint effusion in the right hip (Figure 1).

**Table 1:** Patient’s notable laboratory values on admission

Laboratory Test (serum)	Patient’s Value	Reference Range
Hemoglobin	11.6 g/dL	10 - 13 g/dL
White blood cell	14400 cells/mm³	8000 - 12000 cells/mm³
Neutrophil cell	6500 cells/mm³	3000 - 5000 cells/mm³
C-reactive protein	122 mg/L	< 6 mg/L
Erythrocyte sedimentation	65 mm/hr	< 13 mm/hr

An arthrocentesis of the right hip was performed, yielding purulent fluid, which was sent for bacteriological study. The decision was made to perform an arthrotomy via a Heuter approach, resulting in the drainage of 10 cc of frank pus. Irrigation with betadine saline was performed, and a Delbet drain was placed. The patient was placed in Zenith traction, and empiric dual intravenous antibiotic therapy was initiated, comprising ceftriaxone (75 mg/kg/day) and gentamicin (3 mg/kg/day).

After three days of treatment, the fever resolved, and the patient regained full range of motion in the affected hip; the CRP decreased to 50 mg/L. Microscopic examination of the synovial fluid revealed Gram-negative diplococci; subsequent cultures identified Neisseria meningitidis serogroup W135, which was sensitive to ceftriaxone. An immunodeficiency workup was started, which included tests for HIV, immunoglobulin levels (IgA, IgM, IgG), and complement (C3, C4, and CH50). All results were within normal limits.

Following seven days of intravenous antibiotics, the patient achieved complete recovery, characterized by painless hip range of motion and normalization of C-reactive levels. He was discharged with an oral prescription of cefixime at 15 mg/kg/day. Monthly clinical and biological monitoring, including erythrocyte sedimentation rate tracking, was performed, leading to the discontinuation of antibiotic therapy after six weeks. At the 12-month follow-up, no clinical or radiological complications were observed.

## Discussion

The incidence of pediatric septic arthritis varies between four and 37 per 100,000 patients depending on studies [1]. Staphylococcus aureus is the most frequently implicated pathogen; however, other organisms, such as Escherichia coli, Kingella kingae, and Group B Streptococcus, have also been identified [1,4]. Primary septic arthritis due to Neisseria meningitidis remains extremely rare [2,5,6]. It presents as acute septic arthritis without meningitis, purpura, or signs of meningococcemia. To our knowledge, fewer than 50 cases involving both children and adults have been reported in the literature [3,6].

Meningococcal septic arthritis occurs when N. meningitidis present in the blood colonizes the joint synovium, such as the knee, hip, or wrist; the knee is the most commonly involved joint [2]. Most reported cases involve monoarticular involvement, although polyarticular manifestations have also been reported [7]. Clinically, it is impossible to differentiate primary meningococcal arthritis from other causes of septic arthritis [6]. Furthermore, certain prodromes may precede the clinical presentation, such as respiratory symptoms (50-60%) or a maculopapular erythematous rash (50%) [8,9]. Establishing a definitive diagnosis requires distinguishing primary meningococcal arthritis from several clinical mimics that present with similar joint pain and restricted mobility. Transient synovitis remains the most common differential in children; however, it typically lacks the high inflammatory markers and systemic toxicity associated with a bacterial infection. More serious considerations include acute hematogenous osteomyelitis, which may present with adjacent joint effusion, and tuberculous arthritis, characterized by a more chronic, indolent course and specific radiographic findings. Furthermore, inflammatory conditions like juvenile idiopathic arthritis must be ruled out, particularly when systemic symptoms or multi-joint involvement are present. Given the clinical overlap, clinicians must rely on a combination of synovial fluid analysis, elevated CRP, and advanced imaging to exclude these alternatives and ensure timely surgical or antibiotic intervention. In our case, respiratory symptoms preceded the clinical onset. Synovial fluid culture identified Neisseria meningitidis serogroup W135. A literature review of primary septic arthritis due to N. meningitidis serogroup W135 revealed few articles, confirming the rarity of this condition [2,5,9,10]. Serogroup W135 strains have been significantly associated with meningococcal arthritis [10]. To our knowledge, this is the third globally reported case of primary septic arthritis of the hip caused by Neisseria meningitidis serogroup W135 [5,9]. The diagnosis of this condition is established through synovial fluid culture or molecular studies using real-time (RT)-PCR; it should be noted that RT-PCR is three times more sensitive than culture for identifying N. meningitidis [4,6,8]. Under microscopy and in culture, N. meningitidis and N. gonorrhoeae appear similar, highlighting the importance of PCR to support the diagnosis, particularly in cases of negative cultures [4,11]. In our patient, the diagnosis was confirmed through synovial fluid culture.

Deficiencies in complement factors C3, C4, C5, C6, C7, and C8 have been described as risk factors for meningococcal infection [2,5]. In our patient, the immunological assessment, including HIV serology and measurement of immunoglobulins (IgA, IgM, and IgG) and complement levels (C3, C4, and CH50), was negative. According to reports, meningococcal W is more likely to cause extra-meningeal foci, including arthritis, penicillin resistance, and unusual presentations, all of which may delay diagnosis [12].

The treatment of this type of condition is based on drainage of the effusion combined with antibiotic therapy. This consists of intravenous ceftriaxone for seven days, followed by oral antibiotics for a duration of three to four weeks [2,12,13].

With early diagnosis and appropriate treatment, this condition can resolve favorably without sequelae. Meningococcal vaccination (Men ACYW) [2] can prevent this pathology; however, the national immunization program does not include this vaccine. This report highlights the need to consider meningococci as the causative agent in children presenting with arthritis in the presence of respiratory signs, even in the absence of typical symptoms of meningococcal infection.

While this case provides significant clinical insights, it is subject to certain limitations. First, as a single case report, the findings and clinical course observed may not be generalizable to all pediatric presentations of N. meningitidis W135. Furthermore, our diagnosis relied solely on synovial fluid cultures. The lack of molecular testing, such as RT-PCR, represents a diagnostic limitation; as previously noted, PCR is significantly more sensitive than culture and could have provided a more rapid or definitive confirmation, particularly if antibiotic therapy had been initiated prior to sampling. Nevertheless, the successful isolation of serogroup W135 via culture remains a robust finding.

## Conclusions

While Staphylococcus aureus remains the predominant pathogen in pediatric osteoarticular infections, this study emphasizes that Neisseria meningitidis, specifically serogroup W135, must be recognized as a critical etiological agent. The diagnostic challenge lies in its clinical subtlety; as demonstrated, invasive meningococcal disease can manifest as an isolated bone or joint infection, entirely bypassing classic meningeal signs or purpura. Such atypical presentations necessitate a high index of clinical suspicion to avoid diagnostic delays. Ultimately, maintaining this vigilance is essential for initiating prompt targeted therapy, improving functional outcomes, and preventing complications.
